# Integrative lipidomic features identify plasma lipid signatures in chronic urticaria

**DOI:** 10.3389/fimmu.2022.933312

**Published:** 2022-07-28

**Authors:** Jie Li, Liqiao Li, Runqiu Liu, Lei Zhu, Bingjing Zhou, Yi Xiao, Guixue Hou, Liang Lin, Xiang Chen, Cong Peng

**Affiliations:** ^1^ Department of Dermatology, Xiangya Hospital, Central South University, Changsha, China; ^2^ Hunan Key Laboratory of Skin Cancer and Psoriasis, Xiangya Hospital, Central South University, Changsha, China; ^3^ Hunan Engineering Research Center of Skin Health and Disease, Xiangya Hospital, Central South University, Changsha, China; ^4^ National Clinical Research Center for Geriatric Disorders, Xiangya Hospital, Central South University, Changsha, China; ^5^ BGI-Shenzhen, Shenzhen, Guangdong, China

**Keywords:** chronic urticaria, lipidomic signatures, glycerophospholipid, phosphatidylcholine, mast cell

## Abstract

Chronic urticaria (CU) is a chronic inflammatory skin disease mainly mediated by mast cells. Lipids exert essential functions in biological processes; however, the role of lipids in CU remains unclear. Nontargeted lipidomics was performed to investigate the differential lipid profiles between CU patients and healthy control (HC) subjects. Functional validation studies were performed *in vitro* and *in vivo* including β-hexosaminidase release examination from mast cells and passive cutaneous anaphylaxis (PCA) mouse model. We detected dramatically altered glycerophospholipids in CU patients compared with HCs. Phosphatidylserine (PS), phosphatidylethanolamine (PE), and phosphatidylglycerol (PG) were increased, while phosphatidylcholine (PC) was reduced in CU patients. The reduction in PC was related to a high weekly urticaria activity score (UAS7), while PS was positively associated with the dermatology life quality index (DLQI). We also identified the differential lipid profiles between chronic spontaneous urticaria (CSU), symptomatic dermographism (SD), and CSU coexist with SD. CU patients were classified into two subtypes (subtype 1 and subtype 2) based on consensus clustering of lipid profiling. Compared with patients in subtype 2, patients in subtype 1 had elevated levels of PC (18:0e/18:2) and PE (38:2), and lower urticaria control test (UCT) scores indicated worse clinical efficiency of secondary generation H1 antihistamines treatment. Importantly, we found that supplementation with PC could attenuate IgE-induced immune responses in mast cells. In general, We described the landscape of plasma lipid alterations in CU patients and provided novel insights into the role of PC in mast cells.

## Introduction

Chronic urticaria (CU) is a chronic inflammatory skin disease characterized by wheals, angioedema, or both for more than 6 weeks that seriously affects human health (1). CU is classified into chronic spontaneous urticaria (CSU) and chronic inducible urticaria (CIndU), based on whether the lesions occur spontaneously or are induced by specific factors (2). Symptomatic dermographism (SD) often coexists with CSU and is the most common type of CIndU (2). The pathogenesis of CU, especially CSU, is still not fully understood. The central event of CSU pathogenesis is the degranulation of skin mast cells (MCs) activated by various mechanisms, such as crosslinking of immunoglobulin E (IgE) and high-affinity IgE receptors (FcϵRI). MCs release preformed and preactivated mediators such as histamine, cytokines, and chemokines, which cause vasodilation and extravasation, sensory nerve activation, and cell infiltration (3). Activation of coagulation cascades, genetic susceptibility, and type I (IgE mediated) and type II (IgG or IgM autoantibodies) autoimmune reactions have all been shown to be associated with CSU (3, 4). The pathogenesis of SD is also suggested to be related to MCs (3).

Secondary-generation H1-antihistamines (sgAHs) are the evidence-based first-line therapy for CU. However, the efficacy of this therapy is not very satisfactory. The evidence demonstrated that between 40% and 55% of patients did not respond to conventional sgAHs treatment (5); therefore, it is necessary to investigate potential biomarkers for urticaria treatment response (2). Although our previous studies revealed that adenosine (ADO) was a potential candidate biomarker for predicting the efficacy of nonsedating H1-antihistamines in CSU patients and that *ORAI1* gene SNP rs3741595 is associated with responsiveness to the nonsedating H1 antihistamine desloratadine (6, 7), further studies are needed.

Lipid metabolism, glucose metabolism, and amino acid metabolism play central roles in maintaining normal physiological function and homeostasis. Based on lipid biochemical features, lipids have been classified into eight subtypes: fatty acids, glycerolipids, glycerophospholipids, sphingolipids, and sterol lipids (8, 9). The maintenance of energy is a fundamental function of lipids such as fatty acids and glycerolipids. Some lipids, including glycerophospholipids and sphingolipids, act as secondary messengers to trigger signaling pathways, while sphingolipids, glycerophospholipids, and cholesterol make up the cell membrane (10). Alteration of lipid catabolism has been documented in a variety of diseases, such as lysosomal storage disease, diabetes, obesity, cancer, Alzheimer’s disease, and psoriasis (11–16).

Mast cells are well documented to be one of the innate immune cells that act as the first line of defence against a variety of stimuli between the host and the outside environment (17). Lipid remodeling can directly or indirectly affect MC activation; for example, phospholipase A2 (PLA2) is an enzyme for cleaving cell membrane phosphodiacylglycerides, which induces skin epidermal cell release of lysophosphatidylcholine (LPC), leading to MC reactivity and degranulation (18, 19).

In this study, we performed lipidomics to clarify the lipid profiling of CU patients by nontargeted metabolomics and explore the correlation between lipid characteristics and CU classification, severity, efficacy, etc.

## Methods and materials

### Subjects and sample collection

82 CU patients (39 CSU, 23 SD, and 20 CSU coexist with SD) and 82 Healthy control subjects (HC) matched for age, gender, and body mass index (BMI) values were recruited from the Dermatology Clinic and the Health Examination Center of Xiangya Hospital, respectively (**Table 1**). This study was reviewed and approved by the local Ethics Institutional Review Board (IRB) (Xiangya Hospital, Central South University, IRB-201904112). All subjects gave signed informed consent. The diagnosis of CU was based on the international guidelines for urticaria (20, 21) and clinically evaluated by weekly urticaria activity score (UAS7) scores, dermatology life quality index (DLQI) scores, and urticaria control test (UCT) scores(refer to the **Supplementary Materials**). All enrolled CU patients were uniformly treated with sgAHs monotherapy at licensed doses.

**Table 1:** Demographics of chronic urticaria patients (CU) and healthy control subjects (HC).

Characteristics	CU	HC	P-value
Number	82	82	1
Age in years	36.66 ± 9.96	38.71 ± 9.17	0.173
Gender	29 males, 53 females	29 males, 53 females	1
BMI	22.96 ± 3.73	23.16 ± 3.24	0. 715
Race/ethnicity	100% Chinese	100% Chinese	1
UAS7 score	26.34 ± 13.76	N/A	N/A
DLQI score	4.90 ± 4.77	N/A	N/A
UCT score	12.50 ± 4.04	N/A	N/A

BMI, Body Mass Index; UAS7, Urticaria activity score; DLQI, Dermatology Life Quality Index; UCT, Urticaria Control Test; N/A, Not Applicable. Values are presented as the means ± standard deviation. P-value was calculated by unpaired-T test or unpaired-Wilcoxon test. The level of significance was defined as p < 0.05.

The inclusion criteria for CU patients were as follows: 1) Meeting the diagnostic criteria of CSU and SD (CSU, spontaneous appearance of wheals, angioedema, or both ≥6 weeks due to known or unknown causes; SD, itching and/or burning skin and the development of strip-shaped wheals due to shear force acting on the skin); 2) aged 18-60 years; 3) No gastrointestinal diseases, allergic diseases, autoimmune diseases, metabolic diseases, or other known diseases (such as hypertension and coronary heart disease); 4) Residence in Changsha nearly one year before sample collection.

Exclusion criteria for CU patients were as follows: 1) The presence of other subtypes of urticaria, such as acute urticaria, heat-induced urticaria, etc; 2) The sample cannot be collected as required; 3) Pregnancy or breastfeeding; 4) Took other medications or supplements that may affect lipid metabolism.

Inclusion criteria for HC were as follows: 1) aged 18-60 years; 2) No gastrointestinal diseases, allergic diseases, autoimmune diseases, metabolic diseases, or other known diseases (such as hypertension and coronary heart disease); 3) Residence in Changsha nearly one year before the sample collection.

Exclusion criteria for HC were as follows:1) Failure to collect samples as required; 2) Pregnancy or lactation; 3) Took other medications or supplements that may affect lipid metabolism.

After fasting overnight, 10 ml of whole blood from each subject was collected into ethylene diamine tetraacetic acid (EDTA) tubes and centrifuged at room temperature for 20 minutes at 3100 g. Then, plasma samples were collected and immediately stored at -80° C until use.

### Sample preparation and lipid extraction

Lipids in plasma samples were extracted with isopropanol (precooled at -20°C) spiked in lipid internal standard mix (SPLASH^®^ LIPIDOMIX^®^ Mass Spec Standard, Avanti, USA) as previously reported (13). After vortex for 1 minute and incubating at -20 °C overnight, samples were centrifuged for 20 minutes at 14,000 r.p.m, and the supernatants were transferred to autosampler vials for LC-MS analysis. A quality control (QC) sample was prepared by pooling the same volume of each sample to evaluate the reproducibility of the whole LC-MS analysis.

### The UPLC-MS/MS method

Lipids were analyzed by Qexactive mass spectrometer (Thermo Fisher Scientific, USA) with CSH C18 column (1.7 μm 2.1*100 mm, Waters, USA) for separation. The following gradient was used for elution: 0~2 minutes, 40%~43% of liquid B (10 mM ammonia formate, 0.1% formic acid, 90% isopropyl alcohol, and 10% acetonitrile); 2~2.1 minutes, 43%~50% B liquid; 2.1~7 minutes, 50%~54% B solution; 7~7.1 minutes, 54% ~ 70% liquid B; 7.1~13 minutes, 70% ~99% B liquid with flow rate at 0.35 mL/minutes.

The mass spectrometric settings for positive/negative ionization modes were as follows: spray voltage, 3.8/–3.2 kV; aux gas heater temperature, 350 °C; capillary temperature, 320 °C. The full scan range was 200–2000 m/z with a resolution of 70,000, and the AGC target for MS acquisitions was set to 3e6 with a maximum ion injection time of 100 ms. The top three precursors were selected for subsequent MS fragmentation with a maximum ion injection time of 50 ms and resolution of 17,500, the AGC was 1e5. The stepped normalized collision energy was set to 15, 30, and 45 eV. LipidSearch 4.1 SP2 software (Thermo Fisher, USA) was used for lipid identification and quantitation. Data scaling and normalization were further processed using metaX (22).

### Plasma PC were detected by ELISA

Plasma from age-, gender-, and BMI-matched CU patients (n=42) and HC individuals (n=42) was prepared as previously described (**Table S1**). ELISA kit for PC detection was purchased from Shanghai Jianglai Bio-Technology Co. Ltd. (JL19650, Jianglai, China). The experimental procedure follows the ELISA protocol.

### Cell culture

RBL-2H3 cells (ATCC, Manassas, VA) were cultured in MEM (Biological Industries, Israel) with 15% foetal bovine serum, 100 U/mL penicillin, and 100 mg/mL streptomycin at 37°C with 5% CO2 in a humidified atmosphere.

Bone marrow mononuclear cells (BMMCs) were derived from female C57 mice at approximately 4 to 6 weeks of age. Isolated bone marrow progenitor cells were cultured in RPMI 1640 media (Biological Industries, Israel) supplemented with foetal bovine serum (FBS, 10%), penicillin (100 U/mL), streptomycin (100 mg/mL), L-glutamine (2 mM), sodium pyruvate (1 mM), HEPES (10 mM) and recombinant cytokines (stem cell factor, 20 ng/mL; IL-3, 20 ng/mL) at 37°C with 5% CO2 in a humidified atmosphere. After 4 weeks, cultures were composed mainly of MCs (90.1%), as the survival rate and differentiation rate of BMMCs were determined by the Cytek Dxp Athena flow cytometer. Analysis of acquired data was performed with FlowJo software. Analysis of the stained populations was performed by gating on the single (FSCW versus FSC), live cells (FSC versus DYE). Then identify a specific mast cell population expressing both CD117 and the FcϵR (CD117 versus FC) (**Figure S1**).

### Measurement of degranulation

β-Hexosaminidase (β-hex) release, as a marker of MCs degranulation, was assayed by using a fluorometric assay. The RBL-2H3 cells were plated into 12-well plates (9 × 104 cells per well) and BMMCs were plated into 96-well plates (8 × 103 cells per well). After sensitized with 0.1 µg/mL DNP (dinitrophenylated)-specific IgE (Sigma, USA) overnight, the cells were then treated with or without PC (1, 10, or 100 nmol/mL, P7443, Sigma-Aldrich, USA) for 12 h. Then the PC-containing medium was removed and washed cells with dulbecco’s phosphate buffered saline(D-PBS) twice. To examine degranulation, the cells were stimulated with 1 µg/mL DNP-HSA (dinitrophenylated human serum albumin, Biosearch Technologies, USA) for 30 minutes. Then, cell culture supernatant was collected, and cell pellets were lysed with NP-40 (3 mM). Supernatants and cell lysates, respectively, were incubated with reaction buffer (3 mM p-nitrophenyl-N-acetyl-β-D-glucosaminide, pH 4.4, Sigma, USA) for 1.5 h at 37 °C. The mixture reaction was terminated by 150 µL stop solutions (Na2CO3/NaHCO3, pH 10.6). Then, absorbance at 405 nm was measured with a microplate reader (BIO-RAD Laboratories, USA). The β-hex release was evaluated by using the following formula: β-hex release(%)= [(absorbance of the supernatant)/(optical absorbance of the supernatant+ absorbance of the cell pellet)]×100%.

### Animal experiments

BALB/c mice (6-8 weeks, female) were purchased from Hunan SLAC Experimental Animal Co., Ltd. (Hunan, China) and housed under specific pathogen-free conditions at the Department of Animal Experimental of the Central South University. All animal experiments were conducted by the principles of the Guideline for the Management and Use of Laboratory Animals in China and approved by the Animal Ethics Committee of Central South University. Mice were divided into four groups: 1) blank control group; 2) passive cutaneous anaphylaxis (PCA) model group; 3) PCA +PC intervention group; 4) only PC intervention group. The PCA+PC group and PC treatment group were subcutaneous injections in each ear with 15 ul PC (30 mg/kg/d, P7443, Sigma-Aldrich, USA) for 3 consecutive days and others received an equal volume of saline. DNP-specific IgE (250 ng) was subcutaneously injected on day 4. After 20 h, 100 µl DNP-HSA (1 mg/ml) containing 4% (wt/vol) Evan’s blue dye *via* into the angular vein. 30 minutes later, ears were collected and incubated with formamide at 63°C for 18 h (23). The absorbance was measured at the wavelength of 610 nm (BIO-RAD laboratory, USA). The mice were sacrificed 12 h after DNP-HSA injection. The other ear was collected to be stained with toluidine blue, and RT-qPCR was used to detect inflammatory factors.

### RT-qPCR

Total RNA was extracted from mouse ear tissues using Trizol Re reagent according to the manufacturer’s instructions. The purity and concentration of total RNA were determined by Nanodrop spectrophotometer (ND-2000, Thermo Fisher), and total RNA was reverse transcripted by cDNA Synthesis Supermix. Quantitative polymerase chain reaction mixture (10 µl), consisting of Ultra SYBR mixture (5 µl, low ROX), forward and reverse primers (0.2 µl), and genomic DNA (5 ng). The thermal cycle is performed as follows: 95°C for 10 minutes, then 95° C for 15 seconds, then 60° C for 1 minute, for a total of 40 cycles, and then extended for 5 minutes at 72°C to complete the thermal cycle. The 2^−ΔΔ^CT method was used to analyze the relative changes in cytokine gene expression. The primers were purchased from Sangon Biotech (Shanghai, China) and the sequences were designed as follows: For β-actin, the forward primer was 5’- CTACCTCATGAAGATCCTGACC-3’ and the reverse primer was 5’- CACAGCTTCTCTTTGATGTCAC-3’. For IL-4, the forward primer was 5’-TACCAGGAGCCATATCCACGGATG-3’ and the reverse primer was 5’-TGTGGTGTTCTTCGTTGCTGTGAG-3’. For IL-10, the forward primer was 5’- TTCTTTCAAACAAAGGACCAGC-3’ and the reverse primer was 5’- GCAACCCAAGTAACCCTTAAAG-3’. For IL-13, the forward primer was 5’- CTCTTGCTTGCCTTGGTGGTCTC-3’ and the reverse primer was 5’- GGGAGTCTGGTCTTGTGTGATGTTG-3’. For TNF-α, the forward primer was 5’- ATGTCTCAGCCTCTTCTCATTC-3’ and the reverse primer was 5’- GCTTGTCACTCGAATTTTGAGA-3’. For CCL2, the forward primer was 5’- TTTTTGTCACCAAGCTCAAGAG-3’ and the reverse primer was 5’- TTCTGATCTCATTTGGTTCCGA -3’. For CCL3, the forward primer was 5’- TTGCTGTTCTTCTCTGTACCAT-3’ and the reverse primer was 5’- AATAGTCAACGATGAATTGGCG-3’.

### Statistical analysis

The time series analysis for lipids was applied based on the fuzzy c-means algorithm implemented in the R package (version 2.48.0) Mfuzz (24). The optimized number of clusters was estimated by calculating the minimum centroid distance. Metaboanalyst (25) was used for biomarker analysis. All figures were drawn using corresponding R packages. The statistical analysis was performed using unpaired student t-test and nonparametric test. Data are expressed as means ± standard deviation (SD). The level of significance was defined as p < 0.05. *p < 0.05; **p < 0.01; ***p < 0.001; ****p < 0.0001.

## Results

### Identification of lipid profiles in the plasma of CU

Untargeted lipid profiling of the 164 subjects (82 CU and 82 HC) was performed using a high-resolution LC-MS/MS platform. The identified lipids were used for statistical analysis. All quality control samples were focused together, and the average coefficient of variation (CV) of all quantified lipids was less than 10%, indicating that the reproducibility satisfied the quality control criteria for the following analysis (**Figure S2A**). For all 814 quantified lipids, the distribution of lipid species was further annotated, indicating that the untargeted lipid profiling provides high coverage of the main lipid species in the plasma (**Figure S2B**).

**Figure 1:**
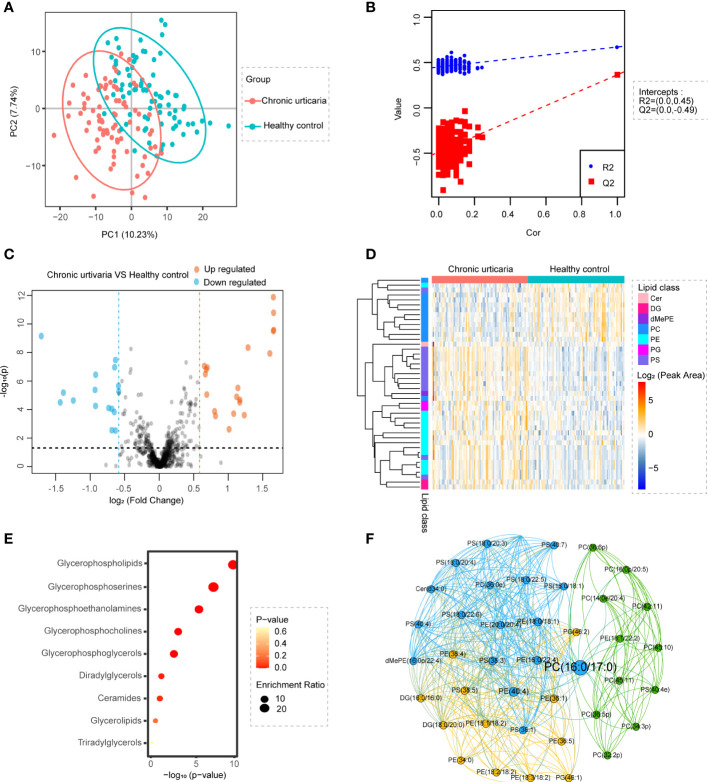
Identification of lipid profiling in plasma of CU. **(A)** PLS-DA score plots show discrimination between the HC (blue) and CU (red). **(B)** Validation plots were obtained from 200 permutation tests (R2 = 0.672, Q2 = 0.365). **(C)** Volcano map of differentially expressed lipids between CU and HC groups. Blue represents the down-regulated lipid molecules, orange represents the up-regulated lipid, and non-significant lipid molecules are gray. Molecules, solid and round represent the lipid molecules VIP ≥1, hollow and round represents the lipid molecules VIP < 1. **(D)** Heatmap of the top 7 differentially lipids between HC and CU. Each row in the figure represents a differential lipid, and each column represents a sample. Different colors indicate different intensities, and colors range from blue to red, indicating strength from low to high. **(E)** Lipid metabolites set enrichment analysis for CU. Redder colors represent lower P values, and larger circles represent higher enrichment ratios. Low P-value and large enrichment ratio indicate that this pathway is enriched more. **(F)** The correlation analysis of different lipids showed that the same lipids had a good correlation.

To explore lipid abundance differences between CU and HC, supervised partial least squares–discriminant analysis (PLS-DA) and t-tests were performed. The screening conditions for differential lipid molecules were as follows: 1) VIP>1; 2) PLS-DA model fold change≥1.5 or ≤0.67; and 3) adjusted p value<0.05. PLS-DA (**Figure 1A**) showed that samples from CU were separated from HC. Cross-validation (**Figure 1B**) 200 times was used to evaluate the PLS-DA model. The R2 value was (0.0, 0.676), and the Q2 value was (0.0, 0.365), indicating that the model was reliable. The total quantification comparison of all species within each major lipid class between HC versus CU is shown in **Figure S2C**. The total quantification of these major lipid classes revealed that phosphatidylserine (PS), phosphatidylethanolamine (PE), and phosphatidylglycerol (PG) in the CU were significantly increased, while the levels of phosphatidylcholine (PC) were significantly decreased. To further determine whether the lipid analysis can differentiate CU patients from HC, a receiver-operator curve (ROC) analysis was performed and found that the AUC could reach 0.8516 (95% CI: 0.7954-0.9078) (**Figure S2D**). The volcano plot (**Figure 1C**) showed that there were 20 upregulated and 16 downregulated lipids in CU subjects compared with HC. All differential lipids were used for clustering, as shown in the heatmap (**Figure 1D**) annotated with lipid species. The upregulated lipid species were mainly PS, PE, and PG, while the downregulated lipid species were mainly PC, which was similar to the functional analysis, as pathways related to glycerophospholipids, glycerophosphoserines, and glycerophosphoethanolamines were enriched (**Figure 1E**), revealing the dysregulation of citydine diphosphocholine (CDP) pathway in CU. The correlation analysis for these differential lipids revealed that the same lipid species shared a similar abundance pattern; for example, all differential PEs correlated well with each PE (**Figure 1F**).

### Alterations in lipids are related to the clinical characteristics of CU

UAS7 and DLQI scores are internationally recognized tools for evaluating the severity of urticaria (26). We assessed CU patients before treatment using UAS7 and DLQI scores. The UAS7 score ranges from 0 to 42, and the higher score reflects the higher activity (27). We divided CU group into four disease states according to the UAS7 score of Khalil et al. (26), including (1) UAS1 (n=6): No urticaria or well-controlled urticaria Activity: 0-6; (2) UAS2 (n=10): Mild Activity: 7-15; (3) UAS3 (n=12): Moderate Activity: 16-27; (4) UAS4 (n=31): Severe Activity: 28-42. Twenty-three patients with SD alone were excluded from this analysis because the UAS7 score was not suitable for assessing disease activity status in patients with SD. The DLQI was used to assess patients’ quality of life on a scale of 0 to 30. The higher the score, the greater the impact of the disease on the Quality of Life of patients (28). We divided CU group into four disease states according to the DLQI score of Khalil et al., including (1) DLQI1 (n=6): No urticaria or well-controlled urticaria Activity: 0-5; (2) DLQI2 (n=10): Mild Activity: 6-10; (3) DLQI3 (n=12): Moderate Activity: 11-20; (4) DLQI4 (n=31): Severe Activity: 21-30.

**Figure 2:**
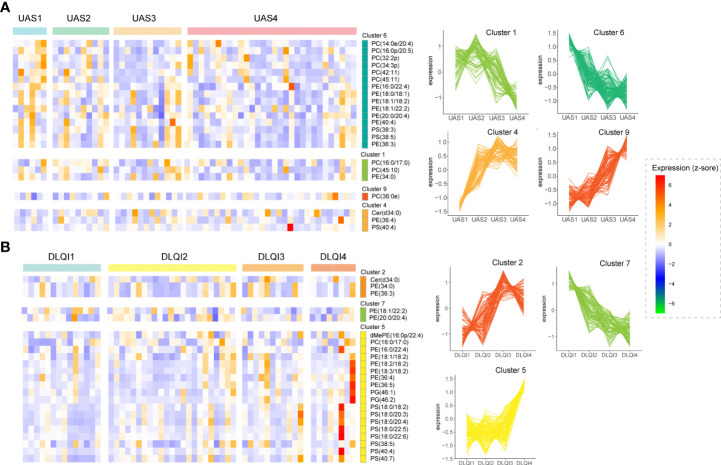
Alterations in lipids are related to the clinical characteristics of CU. Heatmaps of lipid clusters grouped by different UAS7 **(A)** and DLQI **(B)** scores are shown on the left. The abscissa marks the specific scoring groups, and the ordinate marks the cluster and the major lipids in that cluster. The cut squares in the figure correspond to groups and clusters. Clusters with significant trends are on the right.

Cluster analysis was performed on all lipids with CU subgroups using UAS7 or DLQI as series factors. For the UAS7 subgroups, lipids were clustered into 10 specific patterns (**Figure S3A**). Four clusters showed obvious trend changes **(** **Figure 2A**). The abundance of lipids in cluster 6 decreased with higher UAS7 scores, while lipids in cluster 9 increased with higher UAS7 scores. Lipid abundance in cluster 1 decreased in UAS4, while in cluster 4, lipids in UAS1 shared the lowest abundance. Annotation of ratios for different lipid species in these UAS7-related clusters revealed that, as shown in **Figure 2A**, most PCs were enriched in clusters 1 and 6, while PSs and ceramides (Cers) were enriched in clusters 4. For DLQI subgroups, lipids were clustered into 9 specific patterns (**Figure S3B**). Three clusters showed obvious trend changes. The abundance of lipids in cluster 7 decreased with higher DLQI scores, while lipids in cluster 2 increased with higher DLQI scores. Annotation of ratios for different lipid species in these DLQI-related clusters revealed that, as shown in **Figure 2B**, Cer was concentrated in Cluster 2, while most PS was enriched in Cluster 5, among which DLQI4 was the most abundant.

### Identification of lipid signatures in subtypes of CU

**Figure 3:**
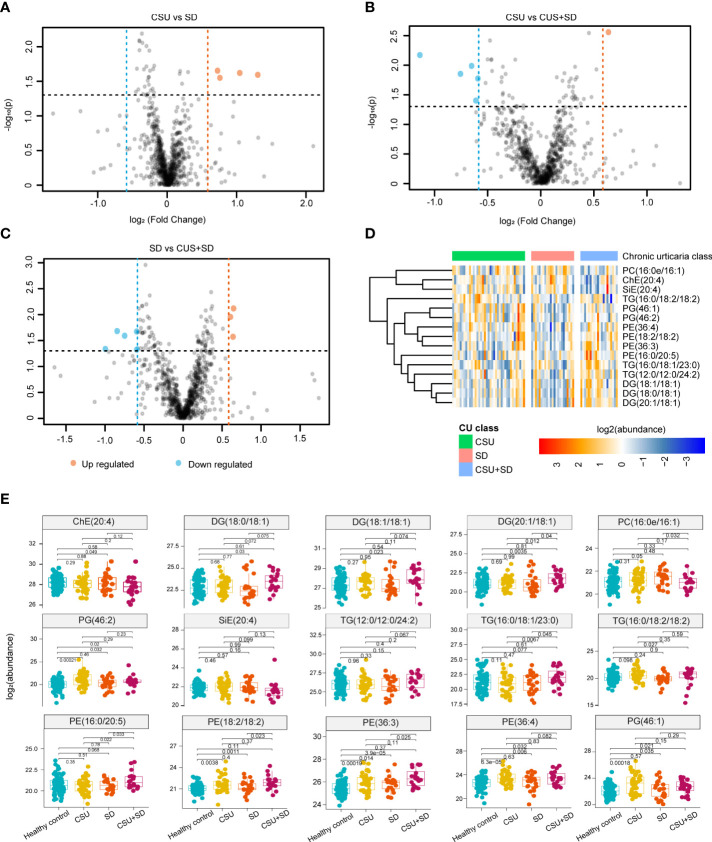
Identification of Lipid signatures in subtypes of CU. **(A-C)** Comparative analysis of lipid abundance between three groups of different types of urticaria as shown in the volcanic diagram. Blue represents the down-regulated lipid molecules, orange represents the up-regulated lipid, and non-significant lipid molecules are gray. **(D)** Heatmap of the top 15 differentially expressed metabolites. Different colors indicate different intensities, and colors range from blue to red, indicating strength from low to high. **(E)** Boxplot of the 15 significantly altered lipids. Blue represents the HC, yellow represents the CU, orange represents the SD, and purple presents the CSU+SD. The y-axis is the normalized intensity after log2 transformation.

We performed an intergroup comparative analysis of lipid abundance in these three groups of different subtypes of urticaria, as shown in the volcano plot (**Figures 3A–C**). We observed that there were 4 upregulated lipids between CSU and SD, 1 upregulated and 5 downregulated lipids between CSU and CSU+SD, and 4 upregulated and 5 downregulated lipids between SD and CSU+SD. The abundance of these 15 lipids is shown in **Figure 3D**. PG was mainly enriched in CSU, PC and cholesteryl ester (ChE) were mainly enriched in SD, while PE and DG were mainly enriched in CSU+SD.

We further analyzed the differential expression of lipids in CU subtypes (CSU, SD, and CSU+SD), and the boxplots of these differential lipids are shown in **Figure 3E**. Compared with HC, PE (18:2/18:2), PE (36:3), PE (36:4), PG (46:1), and PG (46:2) were substantially elevated in CSU, whereas only PE (36:3) and PC (16:0e/16:1) were elevated in SD. Additionally, we found that diacylglycerol (DG) (18:0/18:1), DG (18:1/18:1), DG (20:1/18:1), PG (46:2), and PE (36:3, 36:4) were significantly enriched in CSU+SD, while ChE(20:4) was reduced. Interestingly, we noticed that only the PE (36:3) was altered in CSU, SD, and CSU+SD individuals compared with HC. Moreover, the metabolites of diacylglycerol, such as DG (18:0/18:1), DG (18:1/18:1), and DG (20:1/18:1), were significantly increased in the CSU+SD subtype but were not altered in the CSU or SD subtype, indicating that those metabolites of DG are signatures of CSU+SD.

### Classification of CU lipid subtypes based on profiling

**Figure 4:**
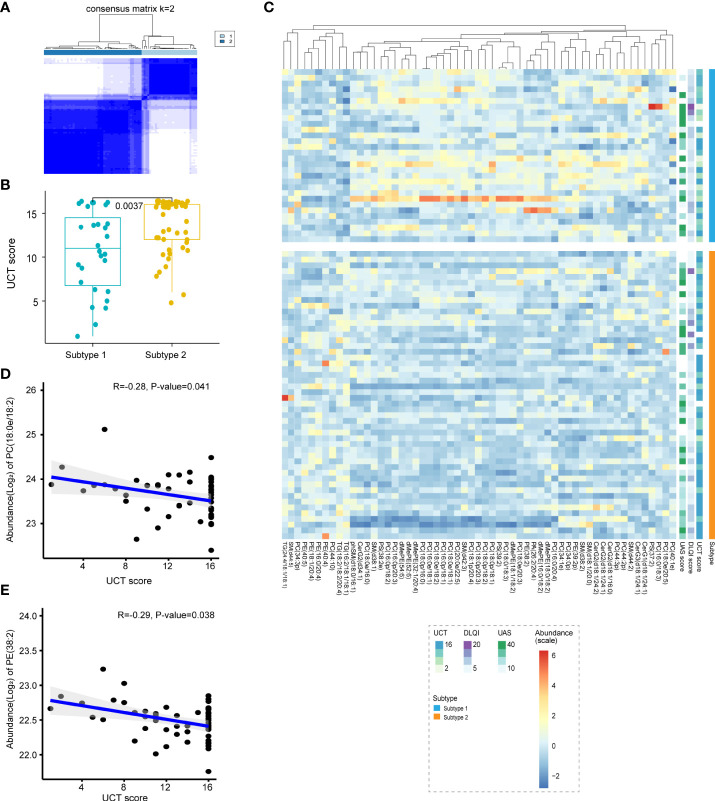
Classification of CU lipid subtypes based on profiling. **(A)** The heat map corresponding to the consensus matrix for 2 metabolite subtypes was obtained by applying consensus clustering. **(B)** CU was classified into two subtypes based on lipid profiling, subtype 2 shared higher UCT scores compared with subtype 1. **(C)** The heatmap of all differential lipids related with subtype 1 (blue) and subtype 2 (orange), which was generated using the heatmap function in R with subtypes, UAS7 (green), DLQI (purple) score and UCT (blue) score as the annotations. The colors range from blue to red, indicating strength from low to high. **(D, E)** Plasma PC (18:0e/18:2) (R=-0.28, P-value=0.041) and PE (38:2) (R=-0.29, P-value=0.038) levels were negatively correlated with UCT scores.

Using unsupervised consensus clustering of lipid-based abundance values, we classified two subtypes of CU, subtype 1 and subtype 2 (**Figure 4A**). Combining lipid-defined CU metabolite subtypes with clinical phenotypes, including UAS, DLQI, and UCT, we found that patients of subtype 2 shared higher UCT scores than those of subtype 1 (**Figure 4B**). UCT score is a scoring tool for clinical evaluation of the therapeutic effect of CU (29), which scores range from 0 to 16 points and higher UCT scores represent well-controlled CU.

Further analysis of the two CU subtypes revealed differential expression of metabolites, including 23 PC, 1 monogylcosylceramide (CerG1), 4 diglycosylceramide (CerG2), 1 triglycosyl-ceramide (CerG3), 3 PS, 6 PE, 3 TG, 6 dimethylphosphatidylethanolamine (dMePE), 6 sphingomyelin (SM), 1 phosphatidic acid (PA) and 1 phytosphingosine (phSM) (**Figure 4C**). Moreover, the findings showed that the abundance of PC (18:0e/18:2) and PE (38:2) was negatively correlated with the UCT score (**Figures 4D, E**), suggesting that they may be potential predictors of the efficacy of H1 antihistamines.

### Supplementation with PC attenuates IgE-induced mast cell activation *in vitro* and *in vivo*

**Figure 5:**
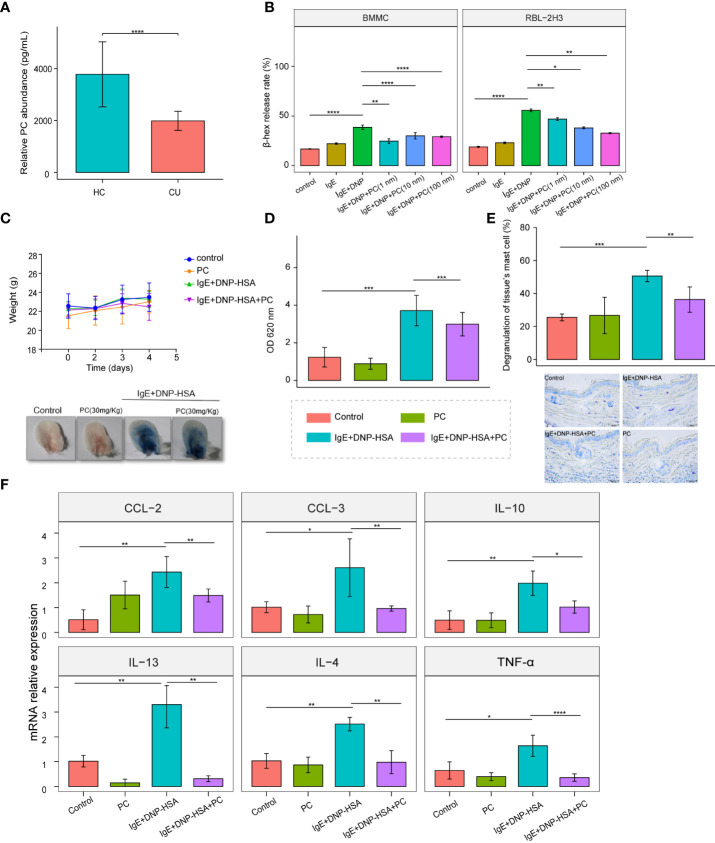
Supplementation with PC attenuates IgE-induced mast cell activation *in vitro* and *in vivo*. **(A)** The mean plasma PC level of the CU patients n = 42 was significantly lower than that of the HC subjects n = 42 were determined by ELISA. **(B)** PC (Including three concentrations: 1 nM,10 nM, and 100nM) inhibited the degranulation of RBL-2H3 cells and BMMCs, and there was no concentration dependence. **(C)** Bodyweight curve and phenotypic changes of four mice groups (Control, n = 5; PC, n = 5; IgE+DNP-HSA, n = 8; IgE+DNP-HSA+PC, n = 8) during the experiment. **(D)** PC intervention reduced skin vascular permeability in the PCA mice model. **(E)** Toluidine blue staining showed that PC inhibited degranulation of skin mast cells. **(F)** The expression of genes involved in cytokine was measured by real- time-qPCR analysis. PC inhibited cytokine release in PCA mice models, including IL-4, IL-10, IL-13, CCL-2, CCL-3, and TNF- α. The data from multiple experiments were expressed as the mean ± standard deviation (SD). Significant differences were evaluated using student t-test or the nonparametric test (Be denoted as follows: ****p≤.0001, ***p ≤.001, **p ≤.01, *p ≤.05).

To validate the reduction of PC in CU, we further performed ELISA to test the abundance of PC in CU patients’ plasma (n=45) as well as HC subjects (n=45). As expected, the concentration of PC was dramatically decreased in CU patients compared with HC subjects (**Figure 5A**).

MCs are a trigger in the pathogenesis of CU; therefore, we examined the role of PC in IgE-induced MC activation. We treated RBL-2H3 cells and BMMCs with different concentrations of PC (1 nM, 10 nM, 100 nM). The results showed that the β-hex release ratio was remarkably downregulated in a dose-independent manner following PC treatment (**Figure 5B**).

Vascular hyperpermeability is a major causative factor of anaphylaxis. The passive cutaneous anaphylaxis (PCA) ear model is a typical mouse model to test IgE-induced MC responses. PC treatment significantly alleviated Evan’s blue dye and vascular permeability of the PCA model (**Figures 5C, D**). Additionally, we found that mast cell degranulation was reduced in the ear tissues of PC-treated mice by toluidine blue staining (**Figure 5E**). It is well known that the degranulation of MCs is accompanied by the release of cytokines and chemokines, which play a major role in the pathogenesis of urticaria. Therefore, we examined the expression of cytokines in the PCA mouse model and found that PC inhibited the expression of IL-4, IL-10, IL-13, CCL-2, CCL-3, and TNF-α at the mRNA level in skin tissues of PCA mice (**Figure 5F**).

## Discussion

Previous studies revealed that CU may be a metabolism-related skin disease (30). Kobayashi previously proposed that the omega-6 and omega-3 series of polyunsaturated fatty acids and lipid peroxidation may be involved in CU pathogenesis (31). Another study identified changes in serum docosahexaenoic arachidonic acid glutamate and succinic acid levels in CSU by serum metabolome analysis, identifying intestinal microbiome-induced unsaturated fatty acid and butyric acid metabolism in CSU patients (32). However, there are few metabolomics studies on CU, and its metabolic characteristics are not fully understood. In our study, plasma lipid characterization in patients with CU was explored by untargeted lipidomics. We found that plasma lipid metabolism features were different in the CU group (including CSU, SD, and CSU+SD) compared with healthy controls, further suggesting the potential role of lipid disturbance in CU.

Glycerophospholipids are the most abundant phospholipids in the body; they form the outer membrane of biofilms and participate in protein recognition and signal transduction by cell membranes. The organization of membrane lipids is a target that regulates mast cell activation: the inner lobule faces inward and contains negatively charged amino phospholipids and PE. The outer lobe faces outwards and contains PC and sphingomyelin (33). The asymmetric distribution of phospholipids on the plasma membrane plays an important role in MC exocytosis (34). A large body of work has been published on the lipid composition of the plasma membrane with proposed partitioning of signaling components into liquid-ordered and liquid-disordered regions (or membrane rafts), including signaling through the FceRI (35). The activity of RBL-2H3 cell surface receptors can be regulated by membrane lipids. Antigen (Ag) crosslinking of the immunoglobulin E receptor (IgE-FcϵRI) complex in mast cells stimulates transmembrane (TM) signaling, requiring phosphorylation of aggregated FcϵRI by lipid-anchored Lyn tyrosine kinases (36). Membrane lipid replacement has been proposed for other chronic diseases such as metabolic syndrome. Membrane lipid substitutes contain a mixture of membrane glycerophospholipids, fatty acids, and other lipids to replace and remove damaged cells and cell intima lipids (37). Abnormal metabolism of glycerophospholipids has also been observed in other diseases, such as asthma (11), osteoporosis (38), psoriasis (39), lupus erythematosus (12), and oesophageal adenocarcinoma (40), but the specific lipid subclasses that change are not the same. In this study, through metabolomic profiling, we found that glycerophospholipids were significantly altered in the plasma of CU patients. The levels of PS, PE, and PG in the CU were significantly increased, while the levels of PC were significantly decreased. PS on the surface of apoptotic cells is thought to be an anti-inflammatory and immunosuppressive signal (41). Free PS and Lyso-PS can enhance FcϵRI-mediated MC degranulation (42). PE, formed by the decarboxylation of PS, acts as an antioxidant and plays an important role in vesicle transport, membrane fusion, and division (43). We hypothesized that PE might be related to the endocytosis and exocytosis of mast cell degranulation. PG has multiple roles in the formation of important lipid-protein and lipid-lipid interactions (10). The role of the PC is described below. These lipids are synthesized and converted through the CDP-DAG pathway (44). The results suggest that the CDP-DAG pathway may be dysregulated in CU.

Consistent with the overall lipid profile of CU, we also found that a higher UAS7 score was associated with a lower plasma concentration of PC, while increased PS expression was associated with an increased DLQI score. The pathogenesis of SD and CSU is not completely clear or identical (3). Our results showed that different subtypes of CU have different lipid profiles. PE (36:3) was found to be CSU specific, and DG metabolites are markers of CSU coexisting with SD. Patients with CSU coexisting with SD often have more severe clinical symptoms than those with CSU or SD alone. These results further indicate that they have different pathogeneses. Interestingly, we found that CU patients could be classified into two subtypes by consensus clustering of differential lipids, subtype 1 and subtype 2, and subtype 2 shares higher UCT scores. The abundance of PC (18:0e/18:2) and PE (38:2) was negatively correlated with the UCT score. This suggests that they can be used as predictors of H1 antihistamine efficacy. Changes in PC and/or PE content in different tissues are associated with metabolic disorders such as atherosclerosis, insulin resistance, and obesity (43). Previous studies indicated that body weight, BMI, and allergic disease could affect the therapeutic efficacy of sgAHs in CU patients (45).

PC, known as anti-inflammatory or antioxidant phospholipids, participates in membrane structure formation and cell signal transduction. PC can improve experimental arthritis and lipopolysaccharide-induced inflammatory responses by regulating leukocyte activation, intestinal brain axis balance, etc (46–49). *In vitro*, PC reduces inflammatory responses in macrophages, monocytes, and Caco-2 cells induced by TNF-α and IL-6, possibly involving inhibition of NF-κB transport activated by MAPKs/ERK and p38 (50, 51). In addition, PC has been proven to have ROS clearance and oxidative stress improvement effects in experimental models of ischaemia and hypoxia (52, 53). In our study, we found that PC could inhibit the mast cell degranulation reaction and reduce the expression of inflammatory factors in the PCA mouse model. The CU is associated with the Th2 immune response (54), and basophils and MCs are major effector cells (3, 55). The expression of the TNF-α, IL-6, and IL-8 genes in MCs depends on the activation of the transcription factor NF-κB (56). NF-κB activation is regulated by MAPK in MCs through a variety of mechanisms, including extracellular signal-regulated kinase (ERK), c-Jun N-terminal kinase (JNK), and p38 mitogen-activated protein kinase (p38) (56). Previous evidence showed that urticaria was mediated by oxidative stress. Plasma oxidative stress increased in CSU patients and was positively correlated with disease activity (57, 58). ROS affects the activation and degranulation of basophils and mast cells (59). Therefore, we speculated that PC may also play a role in alleviating CU inflammation by inhibiting MAPK/ERK and NF-κB activation. Moreover, the antioxidant effect of PC may also affect the occurrence and development of CU. The specific mechanism of PC in CU including investigation of the delivery of PC from the plasma to mast cells needs further research.

**Figure 6:**
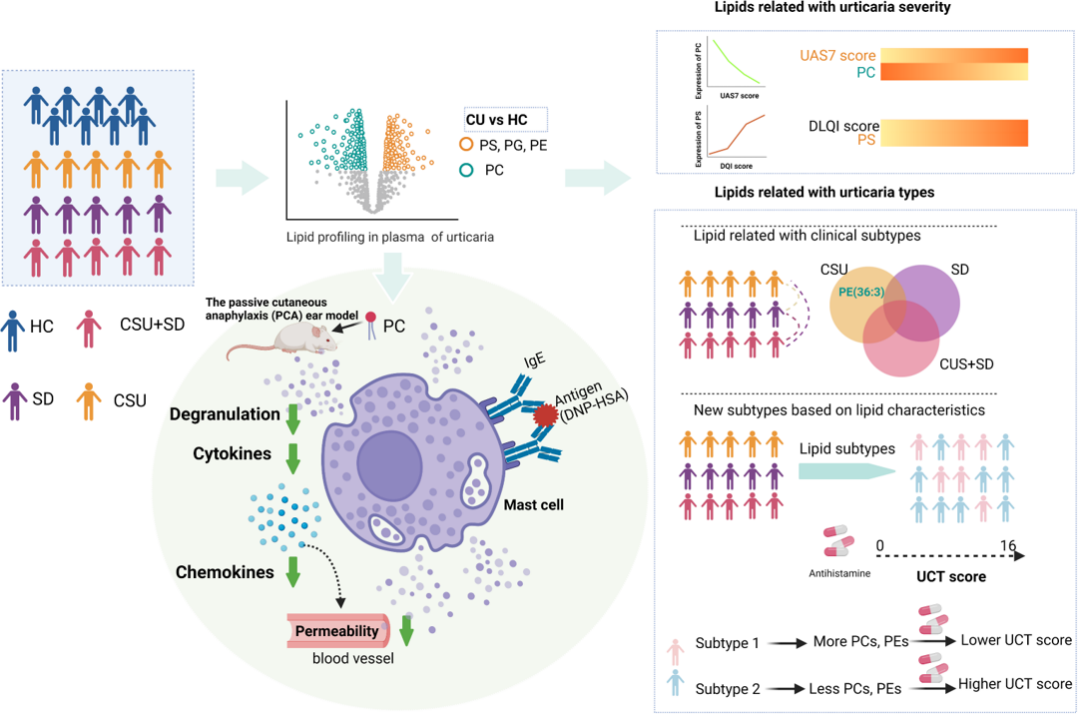
Integrative lipidomic features identify plasma lipid signatures in chronic urticaria. The lipidomics was performed to investigate the differential lipid profiling between CU patients and healthy control subjects (HC). The levels of phosphatidylserine (PS), phosphatidylethanolamine (PE), and phosphatidylglycerol (PG) in CU patients were significantly increased, while those of phosphatidylcholine (PC) were significantly decreased. The decrease of PC was highly correlated with urticaria activity score (UAS7), while PS was positively correlated with dermatological quality of life index (DLQI). Differences in lipid profiles between CSU, SD, and CSU+SD. CU patients were divided into two subtypes, subtype 1 and subtype 2, based on consistent clustering of lipid profiles. Compared with subtype 2, subtype 1 was characterized by increased PC (18:0E/18:2), increased PE (38:2), and lower UCT score, indicating poor clinical efficacy of second-generation H1 antihistamine therapy. PC supplementation attenuated IgE - induced mast cell immune responses in the passive cutaneous anaphylaxis model. This figure is created with https://BioRender.com.

In summary, we employed plasma lipidomics to investigate the potential pathophysiology of CU (**Figure 6**). The results showed that plasma glycerophospholipids in CU patients were significantly changed compared with those in healthy controls. PS, PE, and PG were significantly upregulated, while PC was downregulated. PC has a potential predictive value for disease activity, and PS has a potential predictive value for DLQI. Lipid characteristics distinguish between different subtypes of CU. In addition, CU can be reclassified into two subtypes based on the lipid spectrum, and subtype 2 shares higher UCT scores. PC (18:0e/18:2) and PE (38:2) were negatively correlated with UCT scores, thus indicating their role as predictors of CU antihistamine response. More importantly, we found that supplementation with PC could attenuate IgE-induced immune responses in mast cells, providing a new strategy for the treatment of CU patients.

## Data availability statement

The raw data supporting the conclusions of this article will be made available by the authors, without undue reservation.

## Ethics statement

The studies involving human participants were reviewed and approved by The local Ethics Institutional Review Board (IRB) (Xiangya Hospital, Central South University, IRB-201904112). The patients/participants provided their written informed consent to participate in this study. The animal study was reviewed and approved by The Animal Ethics Committee of Central South University.

## Author contributions

JL, LLi, and CP conceived the project and wrote the manuscript. LLin and GH designed performed experiments and analyzed the data. LLi and BZ helped with mouse experiments. LZ helped with cell experiments. JL, RL, and YX diagnosed patients with chronic urticaria, provided subjects’ samples and information. JL, LLi, and CP edited the manuscript. GH, LLin, XC, and CP supervised the work. All authors contributed to the article and approved the submitted version.

## Funding

This work was supported by National Natural Science, Grant No. 81974476, 82173424, 8213000715, 82073458, 81830096, and by the National Key Research and Development Program of China (2020YFA0112904). The science and technology innovation Program of Hunan Province (2021RC4013), the Program of Introducing Talents of Discipline to Universities (111 Project, No. B20017).

## Conflict of interest

The authors declare that the research was conducted in the absence of any commercial or financial relationships that could be construed as a potential conflict of interest.

## Publisher’s note

All claims expressed in this article are solely those of the authors and do not necessarily represent those of their affiliated organizations, or those of the publisher, the editors and the reviewers. Any product that may be evaluated in this article, or claim that may be made by its manufacturer, is not guaranteed or endorsed by the publisher.
