# Supplementary material for: Integrative lipidomic features identify plasma lipid signatures in chronic urticaria
Source: Front Immunol. 2022 Jul 28;13:933312. doi: 10.3389/fimmu.2022.933312 (PMC9370552; doi:10.3389/fimmu.2022.933312)
Supplement: Supplementary file 1 [file DataSheet_1.docx]

**Supplementary materials**

**Table**

**Table S1: Demographic data of** **chronic urticaria patients (CU) and** **healthy control subjects (HC) for ELISA**

| Characteristics | CU | HC | P-value |
| --- | --- | --- | --- |
| Number | 42 | 42 | 1 |
| Age in years | 38.68±10.08 | 39.12±9.77 | 0.88 |
| Gender | 21 males,21 females | 21 males,21 females | 1 |
| BMI | 24.14±3.93 | 24.55±3.47 | 0.618 |
| Race/ethnicity | 100% Chinese | 100% Chinese | 1 |

BMI, Body Mass Index; Values are presented as the means ± standard deviation. P-value was calculated by unpaired-T test or unpaired-Wilcoxon test. The level of significance was defined as p < 0.05.

**Figure legends**

**Figure S1.** The differentiation rate of BMMCs was detected by flow cytometry. The differentiation rate of mast cells was 90.1% after 4 weeks of culture.

**Figure S2.** (A) PCA score plots of the HC and CU. All quality control samples were concentrated together (the mean coefficient of variation (CV) was less than 10%). (B) A quantitative map of the distribution of identified lipid species, including 814 quantitative lipids. (C) The total quantification comparison of all species within each major lipid class between HC versus CU. (D) The potential value of receiver operating characteristic curve (ROC) analysis based on the differential quantified lipids to differentiate CU patients from HC(AUC: 0.8516, 95% CI: 0.7954-0.9078)

**Figure S3.** Results of k-means cluster analysis of all lipids with CU subgroups using UAS7(A) or DLQI(B) as series factors. For UAS7 subgroups, lipids were clustered into 10 specific patterns. For DLQI subgroups, lipids were clustered into 9 specific patterns. The x-axis corresponds to groups and the y-axis to expression. Different colors distinguish membership.


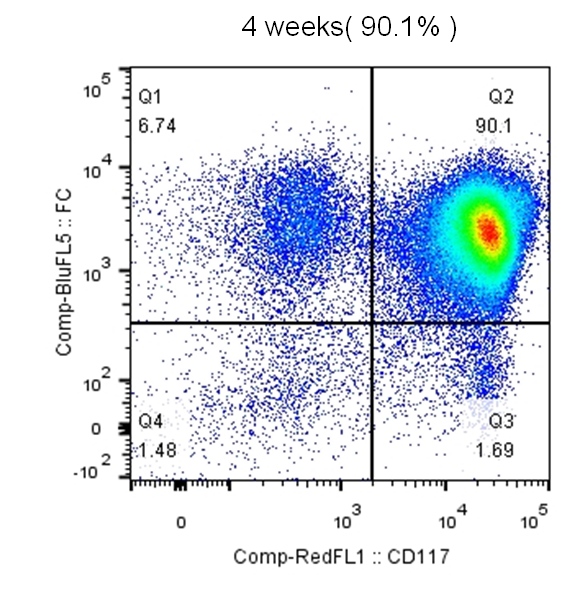


**
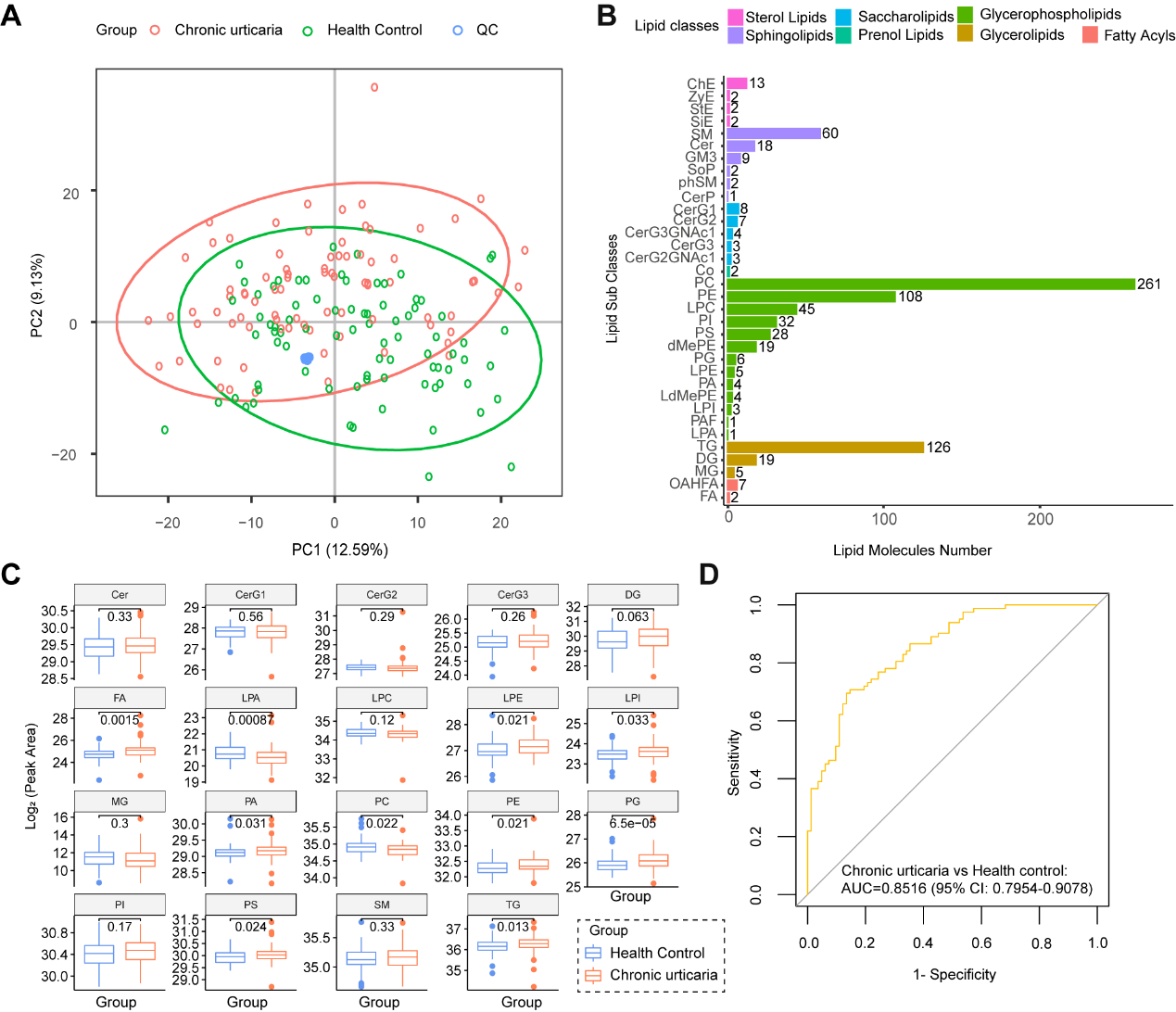
**

**
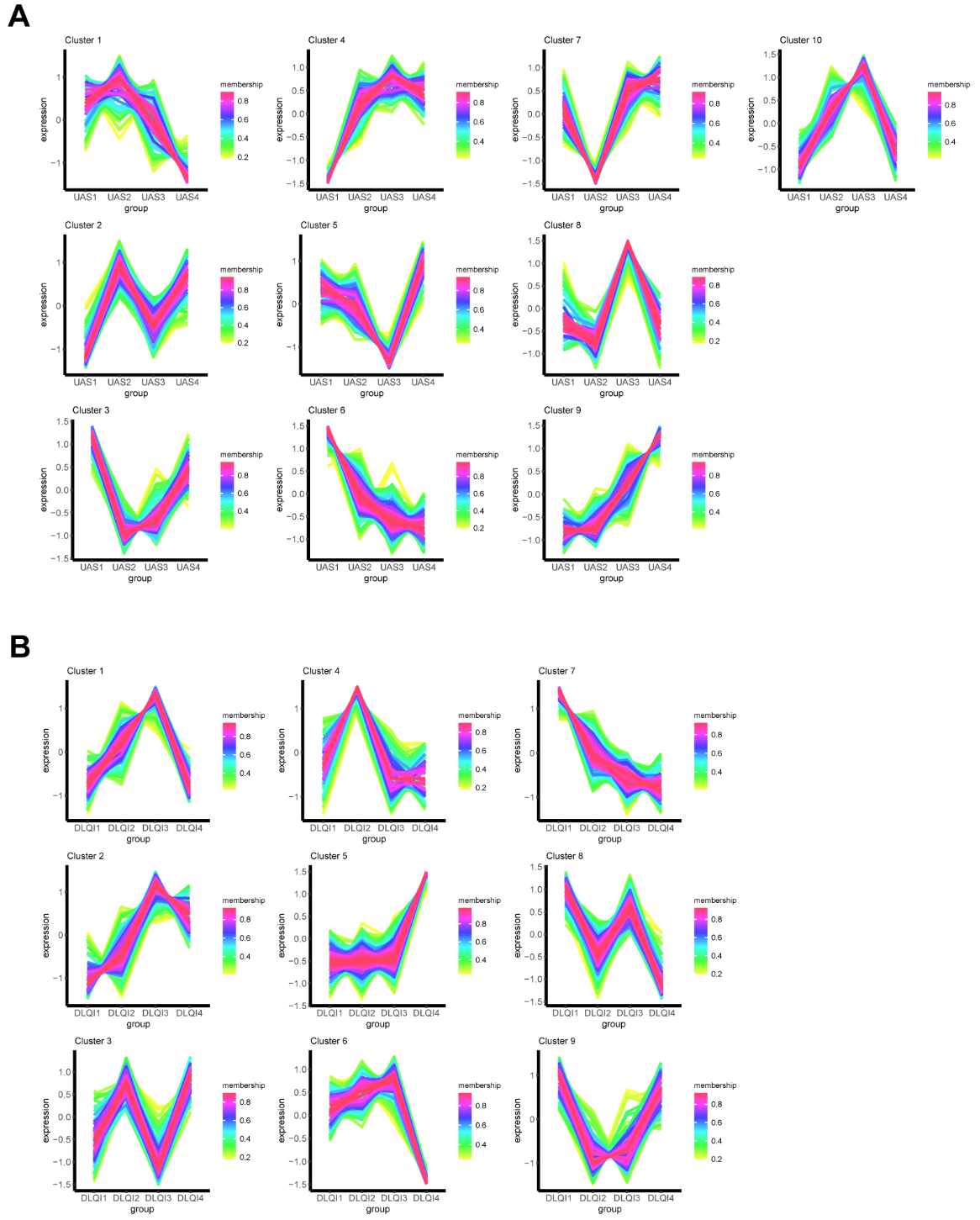
**
